# Gait Biomechanics with Portable EMG Biofeedback at Increasing Muscle-Activation Goals: Walking Speed, Propulsion, Braking, and Step Length

**DOI:** 10.3390/s26165266

**Published:** 2026-08-20

**Authors:** Reza Koiler, Nancy Getchell

**Affiliations:** 1 Biomechanics and Movement Science Interdisciplinary Program, University of Delaware, Newark, DE 19716, USA; 2 Department of Kinesiology and Applied Physiology, University of Delaware, Newark, DE 19716, USA; getchell@udel.edu

**Keywords:** EMG biofeedback, gait, propulsion, walking speed, ground reaction force, wearable sensors, rehabilitation technology

## Abstract

**Highlights:**

**What are the main findings?**

- Higher medial gastrocnemius activation-goal conditions were completed at progressively faster treadmill speeds and were accompanied by graded increases in bilateral propulsion, braking magnitude, and step length.
- Increasing activation goals were associated with changes in stance-phase anterior–posterior and vertical ground-reaction-force profiles without systematic step-length or step-time asymmetry.

**What are the implications of the main findings?**

- Portable auditory EMG biofeedback at increasing activation goals was accompanied by coordinated changes in propulsion-related gait mechanics during a single walking session.
- The graded response across activation goals can guide future studies of individualized feedback difficulty in older adults and individuals after stroke.

**Abstract:**

Portable electromyography biofeedback (EMG-BFB) may support gait rehabilitation, but whole-gait responses across increasing portable auditory feedback goals are unclear. Twenty-four adults completed baseline treadmill walking and four counterbalanced right medial gastrocnemius activation-goal conditions set at 20%, 40%, 60%, and 80% above baseline; 23 contributed primary biomechanical data. Treadmill speed was adjusted within each condition to support achievement of the activation goal. Outcomes included walking speed, ground-reaction forces, force-time metrics, step length, temporal measures, and asymmetry. Repeated-measures MANOVA, outcome-specific repeated-measures ANOVAs, dose-response coefficients, bootstrap intervals, and leave-one-participant-out analyses were used. The combined gait-biomechanics outcomes differed significantly across activation-goal conditions (*p* < 0.001), with large condition effects for walking speed, propulsion, braking magnitude, and step length. From baseline to the highest goal, treadmill speed increased from 1.07 to 1.44 m/s, mean propulsion by 0.086 N/BW, braking magnitude by 0.109 N/BW, and mean step length by 0.150 m. Exploratory speed-adjusted models retained anterior–posterior and vertical loading-response associations but not peak propulsion; activation goal and achieved speed were strongly collinear. Unilateral feedback was not associated with systematic step-length or step-time asymmetry. Portable auditory EMG-BFB at increasing activation goals was accompanied by coordinated changes across gait mechanics.

## 1. Introduction

Walking speed is a clinically meaningful indicator of mobility, community ambulation, health, and independence in older adults and individuals after stroke [1,2,3,4,5]. Although walking speed is often treated as a single global measure, it reflects interacting temporal, spatial, kinetic, and neuromuscular components of gait [6,7,8,9].

One important mechanical component of walking speed is anterior–posterior ground reaction force [6,10]. During late stance, plantar-flexor activity contributes to forward propulsion and redirection of the body’s center of mass. In older adults and individuals following neurologic injuries such as stroke, reduced plantar-flexor contribution, reduced paretic propulsion, and altered trailing-limb mechanics are important contributors to slower walking [8,9,11,12]. Walking speed alone, however, cannot determine whether faster gait reflects increased paretic-limb output or greater reliance on compensatory nonparetic-limb mechanics. Paretic propulsion and interlimb symmetry therefore provide complementary information about the motor strategy underlying changes in post-stroke walking performance [9,13,14]. For this reason, plantar-flexor activity and propulsion are common targets for gait rehabilitation.

Electromyographic biofeedback (EMG-BFB) is one approach for increasing plantar-flexor activation during walking. Prior work has shown that EMG-BFB and propulsion-related gait biofeedback can modify lower-limb activity, gait training outcomes, and propulsion-related gait mechanics in older adults and individuals after stroke [15,16,17,18,19,20,21,22]. However, many EMG-BFB systems require laboratory-grade hardware, wired sensors, real-time signal-processing expertise, and visual displays that are difficult to integrate into natural gait training. These translation barriers matter because the long-term value of gait biofeedback depends on whether it can be delivered in clinical, home, and community settings [23,24,25,26]. Portable systems that can deliver feedback without laboratory infrastructure therefore require validation before they are used as gait-retraining tools.

mTrigger (mTrigger LLC, Newark, DE, USA) is a cost-effective portable EMG-BFB system that was previously developed and validated for gait rehabilitation applications [27]. The platform is a two-channel surface-EMG biofeedback device that connects to a mobile application through Bluetooth and provides real-time feedback from user-selected muscle activity. In the validation study, 32 adults walked overground and on a treadmill at 0.3, 0.6, 0.9, and 1.2 m/s while wearing mTrigger and a laboratory-grade Delsys Bagnoli two-channel EMG system (Delsys Inc., Natick, MA, USA). mTrigger demonstrated good-to-excellent agreement for treadmill step detection and moderate-to-good agreement during overground walking [27]. That work also added gait-specific functions for walking, including auditory feedback, step feedback, activation-goal calibration, success-rate output, and cloud integration. Auditory feedback was selected for the present walking protocol because visual information contributes to foot placement, environmental scanning, and obstacle negotiation during gait [28,29]. Feedback that does not require participants to monitor a visual display may therefore reduce gaze diversion during walking and may be preferable in future overground applications where missed environmental hazards, falls, and trips are relevant safety concerns.

The present study characterized gait biomechanics across increasing right medial gastrocnemius activation goals delivered using portable auditory EMG-BFB during treadmill walking. Activation goals were set at 20%, 40%, 60%, and 80% above baseline medial gastrocnemius activity. We hypothesized that meeting higher activation goals would require progressively faster treadmill speeds and would be accompanied by greater forward propulsion and step length. Because feedback was delivered from only the right limb, we also hypothesized that unilateral feedback might alter step-length or step-time asymmetry.

## 2. Materials and Methods

### 2.1. Participants

Twenty-four healthy adults (mean age 25.4 ± 5.1 years; 10 male, 14 female; body mass 74.9 ± 18.8 kg) participated in the study. The primary gait analysis included 23 participants because one participant’s force data were not fully recorded. Participants were included if they could walk without an assistive device and had no recent musculoskeletal, spinal, or concussion-related injury. Exclusion criteria included a history of gait disorder, neurologic impairment, cardiovascular disease limiting exercise, or other conditions that would make treadmill walking unsafe.

### 2.2. Instrumentation

Participants walked on an instrumented split-belt treadmill (Bertec Corp., Columbus, OH, USA) that recorded ground reaction forces from both belts. Skin over the right medial gastrocnemius was prepared before electrode placement. Excess hair was removed, the skin was cleaned with alcohol wipes, and mTrigger and Delsys electrodes were placed over the medial gastrocnemius according to SENIAM recommendations [30]. The medial gastrocnemius was selected because it contributes to push-off and has been used as a biofeedback target in prior gait studies.

mTrigger was paired with an Android smartphone using Bluetooth. The system includes a portable biofeedback unit, sensing cables, surface electrodes, and a mobile software application. The hardware provides Channel 1 and Channel 2 EMG input ports, supports single- and dual-channel use, and can be used with iOS or Android devices that support Bluetooth Low Energy [31]. The mTrigger gait application had previously been developed to provide real-time step feedback, activation-goal calibration, success-rate tracking, cloud upload, and auditory feedback during walking [27]. In the present study, only the right medial gastrocnemius channel was used to drive auditory feedback. The phone was placed near the participant so the feedback tone could be heard during treadmill walking. Delsys EMG was collected using a custom LabVIEW program (National instruments Corp., Austin, TX, USA), and ground reaction forces were collected during each walking condition.

### 2.3. Activation-Goal Protocol

Participants first completed an overground 10-m walk test to determine self-selected walking speed. This value was used as the initial treadmill speed. Participants then walked on the treadmill for five minutes to allow gait to stabilize. At the end of this baseline period, right medial gastrocnemius activity was used to calculate the baseline mTrigger threshold. Activation goals were then set at 20%, 40%, 60%, and 80% above baseline activity.

Participants were instructed on the role of push-off and plantar flexors during gait. They were asked to “push off harder with the right leg” and “extend the ankle more vigorously” to generate auditory feedback. They were also instructed to focus on pushing backward rather than simply increasing vertical force. After participants were able to elicit feedback, they completed treadmill walking trials at each activation-goal level.

During the familiarization period for each activation-goal condition, treadmill speed was adjusted according to participant comfort and the app-displayed success rate, with at least 80% success used as the intended target for selecting the two-minute walking speed. Participants rested for five minutes after each trial to reduce fatigue and carryover. The four activation-goal trials were counterbalanced using a repeated 4 × 4 Latin-square design: ABCD, BCDA, CDAB, and DABC. Because belt speed was not held constant across activation-goal conditions, the study design characterizes an integrated activation-goal walking state rather than a speed-independent effect of isolated medial gastrocnemius activation.

### 2.4. Biomechanical Outcomes

Outcomes included walking speed, peak right propulsion force, peak left propulsion force, peak right braking force, peak left braking force, step time, stance time, swing time, step length, step length asymmetry, and step time asymmetry. Peak forces were normalized to body weight. Stance time was calculated from heel strike to toe-off of the same limb. Swing time was calculated from toe-off to heel strike of the same limb. Step time was calculated from ipsilateral heel strike to contralateral heel strike. Step length was calculated as the distance from ipsilateral heel strike to contralateral heel strike. Exploratory variability outcomes included within-condition coefficients of variation for selected step-level temporal and force measures.

Step length and step time asymmetry were analyzed to determine whether unilateral right-limb feedback altered gait symmetry. The signed symmetry index was calculated using the difference between limbs normalized to their mean [32,33]:

200 × (Right − Left)/(Right + Left)

Zero indicates symmetry. Positive values indicate larger right-limb values, and negative values indicate larger left-limb values. Because signed asymmetry depends on limb order and the selected reference formulation, direction was retained throughout interpretation [34].

### 2.5. Data Processing

Ground reaction force data were processed using custom MATLAB code (Version 2020b, MathWorks, Inc., Natick, MA, USA). Force data were filtered using a third-order low-pass Butterworth filter applied once, with an effective −3 dB cutoff of approximately 23.6 Hz. A 20 N threshold was used to identify heel-strike and toe-off events. Local step-level outliers in the primary processing were identified using a 10-observation moving-median window and replaced by spline interpolation, after which the first and last two observations were omitted before condition means were calculated. For the added force-time analyses, values outside 1.5 interquartile ranges within each participant-condition-limb cell were omitted before condition means were calculated. Gait-event outputs were screened for physiologic plausibility before temporal-spatial and force-derived analyses. One participant’s force-derived biomechanical data were excluded, and one condition-specific right-limb temporal record produced a negative swing-time value and was excluded as physiologically impossible; right-limb stance and swing analyses therefore included 22 participants. Baseline walking values were subtracted from activation-goal values when change from baseline was analyzed.

The Delsys medial gastrocnemius channel was trimmed by 100 samples at each end and demeaned. The signal was band-pass filtered from 20 to 500 Hz using a zero-phase sixth-order Butterworth filter, full-wave rectified, and then low-pass filtered at 10 Hz using a zero-phase fourth-order Butterworth filter. EMG was normalized within each participant as active-condition amplitude divided by medial gastrocnemius amplitude during the self-selected walking trial. The EMG analysis used the same 23 participants as the primary gait analysis.

Ground-reaction-force profiles were time-normalized to 0–100% of the gait cycle and normalized directly to recorded body weight. Windowed force-profile measures were calculated to characterize early-stance braking and loading, midstance support, and late-stance propulsion because discrete peak values alone may miss important phase-specific changes in the force waveform [35,36]. The windows were selected from established stance-phase mechanics and the expected timing of force peaks in healthy treadmill walking. Early loading response and braking were characterized from 0–15% and 5–25% of the gait cycle, midstance from 20–40%, and terminal-stance propulsion from 35–60%. These windows separated load acceptance, braking, support, and push-off without selecting isolated peaks outside their gait phase. Baseline waveform and baseline-to-activation-goal force-time summaries included 22 participants because one otherwise retained participant lacked a usable raw baseline force recording. Active-condition summaries included 23 participants.

The phase-specific GRF measures were selected to represent complementary biomechanical functions. The anterior–posterior braking peak reflects early stance deceleration and control of forward body progression, whereas the anterior–posterior propulsion peak reflects terminal-stance push-off and forward progression, a commonly used target and outcome in propulsion-focused gait rehabilitation [6,9,10,20,21]. The vertical impact and loading peak, early vertical loading slope, and timing of the first vertical peak describe load acceptance and the rate and timing of vertical support after initial contact, which are often used to interpret how walking speed, step length, and stance mechanics alter lower-limb loading [35,37,38]. The side-normalized mediolateral outward peak was included as a secondary measure of early-stance lateral loading, because a unilateral feedback task could theoretically alter how participants accepted weight or shifted force laterally. Together, these GRF measures tested whether higher EMG-BFB goals primarily increased push-off or produced a broader stance-phase reorganization across braking, loading, support, and propulsion.

Measures included anterior–posterior braking peak from 5–25% gait cycle, anterior–posterior propulsion peak from 35–60%, vertical impact/loading peak and early vertical loading slope from 0–15%, timing of the first vertical peak from 5–25%, and vertical midstance minimum from 20–40%. The early vertical loading slope was defined as the maximum numerical gradient over 0–15% of the participant-condition mean waveform on the one-percentage-point normalized grid. The normalized-waveform early vertical loading slope is reported in N/BW per percentage point of the gait cycle. Raw force-time series sampled at 2000 Hz were used to calculate anterior–posterior braking, propulsive, and net impulses; vertical stance impulse; mediolateral outward impulse; average and peak vertical loading rates; and anterior–posterior braking and propulsion development rates. Impulses are reported in N/BW·s and time-based rates in N/BW/s. The rates describe external force development, not muscle rate of force development. Positive anterior–posterior force represented propulsion and negative force represented braking. Vertical force was positive during stance. Mediolateral profiles were side-normalized using the observed polarity of the two belts so that the dominant outward stance deflection was positive for both limbs. Mediolateral outcomes were interpreted as secondary outward-loading measures.

### 2.6. Statistical Analysis

The study design included four activation-goal conditions and four possible condition orders. A repeated-measures multivariate GLM/MANOVA was used as the primary omnibus analysis, with the activation-goal condition as the within-participant factor and the Latin-square order as the between-participant factor. The multivariate model included walking speed; left and right braking, propulsion, step time, and step length; left stance and swing time; and signed step-length symmetry index. Right stance and swing time were evaluated separately using the 22 participants with complete physiologically plausible values. The MANOVA evaluated the four active-condition change-from-baseline scores. Outcome-specific ANOVAs evaluated the five raw conditions. The step-time symmetry index was not entered separately because it was mathematically equivalent to step-length symmetry index in these treadmill data. Wilks’ Lambda was used for the multivariate condition, order, and condition-by-order tests. The reported outcome-specific F tests were simple five-condition repeated-measures ANOVAs. Greenhouse–Geisser-adjusted *p* values were used as sensitivity analyses when sphericity was violated, and order-aware condition-by-order models were evaluated separately. Bonferroni correction was used for planned pairwise comparisons. Partial eta-squared quantified outcome-specific condition-effect magnitude and was interpreted using conventional thresholds of approximately 0.01, 0.06, and 0.14 for small, medium, and large effects, respectively [39,40]. Cohen’s dz was interpreted using thresholds of 0.20, 0.50, and 0.80 for small, medium, and large paired effects.

Activation-goal dose-response coefficients were calculated for each participant across the 20%, 40%, 60%, 80% feedback levels (MTR20, MTR40, MTR60, and MTR80) and are reported per 20-percentage-point increase in activation goal. Sequence-specific coefficients were calculated from baseline through MTR80. For outcomes with significant condition-by-order interactions, Tukey-adjusted post hoc comparisons were used to compare the sequence-specific coefficients calculated from baseline through MTR80. Thus, a walking-speed coefficient of 0.100 m/s indicates that each higher goal level was associated, on average, with approximately 0.10 m/s faster walking. Positive coefficients for speed, propulsion, step length, or vertical loading indicate progressively larger values at higher activation goals, whereas negative anterior–posterior braking coefficients indicate progressively greater braking because braking force is negative. Assumptions were evaluated using residual and pairwise-difference normality and sphericity checks. Friedman and Wilcoxon signed-rank tests were used as nonparametric sensitivity analyses, and 5000 participant-level bootstrap samples were used for coefficient confidence intervals. Each dose-response test was repeated after sequentially omitting one participant as an influence check. Bonferroni correction was reserved for planned primary pairwise comparisons. Benjamini–Hochberg false-discovery-rate correction was applied separately within the declared exploratory GRF force-time families because each family contained correlated descriptors of the same stance-phase response [41]. Speed-adjusted phase-by-direction models used nonoverlapping gait-cycle bins of 0–14% for loading response, 15–34% for midstance, 35–49% for terminal stance, and 50–64% for pre-swing. These bins avoided overlap between model outcomes and were distinct from the overlapping windows used for the peak and window measures. The models included 114 participant-condition observations from 23 participants and specified participant fixed effects, activation goal scaled per 20-percentage-point increase, and achieved speed as a continuous predictor. Partial R2 was calculated from the reduction in residual sum of squares when activation goal was added to the participant-and-speed model. Inference used participant-clustered covariance, 5000 participant-level bootstrap intervals, partial R2, and leave-one-participant-out influence checks. False-discovery-rate correction was applied across the 12 activation-goal terms.

Primary force and gait processing was performed in MATLAB. Data analysis, sensitivity testing, plotting, and secondary force-profile summaries were performed in Python 3.12.13 using NumPy 2.4.6, pandas 3.0.3, SciPy 1.17.1, statsmodels 0.14.6, and Matplotlib 3.11.0. Statistical tests from these packages and IBM SPSS Statistics for Windows, Version 27 (IBM Corp., Armonk, NY, USA) were used to obtain the repeated-measures findings, estimate activation-goal dose-response coefficients, calculate bootstrap intervals, perform nonparametric sensitivity tests, apply exploratory false-discovery-rate correction, and generate the manuscript figures.

Exact participant, activation-goal, and trial matches were used to compare mTrigger average activation peaks with Delsys amplitude. Spearman rank associations were calculated after within-participant centering because the two systems used different processing and measurement scales. Uncertainty was estimated using 5000 participant-cluster bootstrap resamples.

## 3. Results

### 3.1. Multivariate and Assumption Checks

Repeated-measures MANOVA showed a significant multivariate effect of activation-goal condition on gait biomechanics (Wilks’ Lambda = 0.020, F(36, 136.640) = 10.52, *p* < 0.001). The order main effect was not significant (Wilks’ Lambda = 0.089, F(36, 24.365) = 0.86, *p* = 0.670). The condition-by-order interaction was significant (Wilks’ Lambda = 0.076, F(108, 347.406) = 1.36, *p* = 0.019) and was examined further in order-stratified analyses.

After the primary force-sample exclusion, the four Latin-square sequence groups included 6, 6, 6, and 5 participants for ABCD, BCDA, CDAB, and DABC, respectively. Mean walking-speed coefficients calculated from baseline through MTR80 were positive in every sequence (0.0930, 0.0908, 0.1102, and 0.0808 m/s per 20-point increase), and every participant showed a positive speed direction. Propulsion, braking magnitude, and step length retained the same group-mean direction in all sequences. The condition-by-order interactions were significant for walking speed (*p* < 0.001) and step time (*p* = 0.015) but not for propulsion (*p* = 0.930), braking magnitude (*p* = 0.431), or step length (*p* = 0.291). Post hoc comparisons showed that CDAB produced a greater increase in walking speed than DABC (mean coefficient difference = 0.0294 m/s per 20-point increase, Tukey-adjusted *p* = 0.011) and a greater reduction in step time (mean coefficient difference = 0.0155 s per 20-point increase, Tukey-adjusted *p* = 0.036). CDAB did not differ significantly from ABCD or BCDA for either outcome, and no other pairwise differences were significant. The statistically supported sequence dependence was therefore primarily temporal.

Assumption diagnostics showed that several outcomes violated sphericity and/or normality assumptions. For the primary gait outcomes, Greenhouse–Geisser-adjusted *p* values, Friedman tests, Wilcoxon signed-rank tests, bootstrap confidence intervals, and leave-one-participant-out analyses were used to evaluate the planned repeated-measures findings. The main findings for speed, propulsion, braking magnitude, and step length remained significant across these checks, whereas step-length and step-time asymmetry remained nonsignificant. Condition effects were large for walking speed, step length, mean propulsion, braking magnitude, and step time (partial eta-squared = 0.449–0.955), with the largest effects observed for walking speed and step length. Exploratory GRF impulse and loading-rate findings are reported with Benjamini–Hochberg-adjusted *p* values within their declared families.

### 3.2. Walking Speed

Achieved treadmill speed increased across activation-goal conditions. Mean speed increased from 1.070 ± 0.106 m/s at baseline to 1.443 ± 0.123 m/s at MTR80. Baseline-adjusted speed increased by 0.075 m/s at MTR20, 0.167 m/s at MTR40, 0.270 m/s at MTR60, and 0.374 m/s at MTR80. The five-condition repeated-measures test was significant (F(4, 88) = 465.63, *p* < 0.001, partial eta-squared = 0.955; Friedman *p* < 0.001). The dose-response coefficient was 0.100 m/s per 20-percentage-point increase in activation goal (bootstrap 95% CI: 0.093 to 0.108 m/s; t(22) = 23.77, *p* < 0.001, dz = 4.96). This result describes the achieved speed progression within the individualized activation-goal protocol (Table 1).

### 3.3. EMG Response and Cross-System Association

Delsys medial gastrocnemius amplitude normalized to participant-specific baseline increased across activation-goal conditions (MTR20: 1.722 ± 0.959; MTR40: 1.746 ± 1.032; MTR60: 2.025 ± 1.247; MTR80: 2.227 ± 1.290; F(3, 66) = 6.67, *p* < 0.001, partial eta-squared = 0.233). The baseline-normalized dose-response coefficient was 0.179 per 20-point goal increase (bootstrap 95% CI: 0.074 to 0.316; t(22) = 2.81, *p* = 0.010, dz = 0.587), with substantial between-participant variability. Across 143 synchronized trial pairs from 20 participants, mTrigger activation peaks and Delsys medial gastrocnemius amplitudes were moderately positively associated within participants (Spearman ρ = 0.594, participant-cluster bootstrap 95% CI: 0.382 to 0.755), suggesting that the portable signal broadly reflected the same direction of change as the laboratory EMG. The two systems therefore provide complementary rather than interchangeable measures of muscle activation.

### 3.4. Propulsion and Braking

Peak propulsion increased in both limbs despite feedback being provided from only the right medial gastrocnemius. Left peak propulsion increased from 0.172 ± 0.028 N/BW at baseline to 0.254 ± 0.052 N/BW at MTR80. Right peak propulsion increased from 0.180 ± 0.031 N/BW to 0.271 ± 0.062 N/BW. Mean propulsion showed a significant five-condition effect (F(4, 88) = 73.24, *p* < 0.001, partial eta-squared = 0.769). The activation-goal dose-response coefficient for mean propulsion was 0.020 N/BW per 20-percentage-point increase in feedback goal (bootstrap 95% CI: 0.016 to 0.025 N/BW; *p* < 0.001).

**Figure 1:**
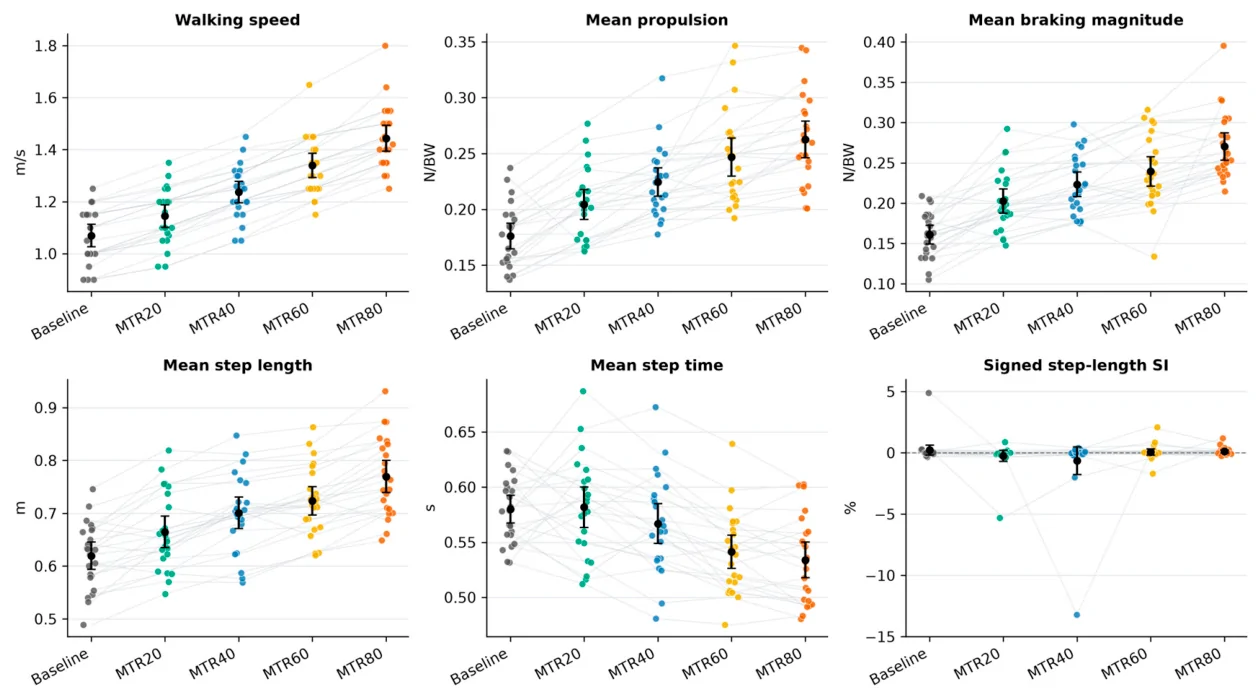
Primary biomechanical response across activation-goal conditions. Dots show individual participants, gray lines connect repeated observations within participants, and black markers show condition means with 95% confidence intervals. Propulsion and braking are shown as mean bilateral peak measures; signed step-length symmetry index is shown across the full observed range. All panels include 23 participants.

Braking magnitude also increased. Mean braking magnitude increased from 0.161 ± 0.028 N/BW at baseline to 0.270 ± 0.042 N/BW at MTR80, with a significant five-condition effect (F(4, 88) = 68.89, *p* < 0.001, partial eta-squared = 0.758). The activation-goal dose-response coefficient was 0.022 N/BW per 20-percentage-point increase in feedback goal (bootstrap 95% CI: 0.017 to 0.026 N/BW; *p* < 0.001). These findings indicate that higher activation-goal walking involved larger anterior–posterior force excursions in both braking and propulsion phases (Figure 1).

### 3.5. Step Length and Temporal Measures

Step length increased with higher activation goals. Mean step length increased from 0.620 ± 0.063 m at baseline to 0.770 ± 0.074 m at MTR80. The five-condition repeated-measures test was significant (F(4, 88) = 102.57, *p* < 0.001, partial eta-squared = 0.823; Friedman *p* < 0.001). The activation-goal dose-response coefficient was 0.034 m per 20-percentage-point increase in feedback goal (bootstrap 95% CI: 0.030 to 0.038 m; t(22) = 15.44, *p* < 0.001, dz = 3.22).

Temporal changes followed the faster-walking pattern. Mean step time decreased from 0.580 ± 0.031 s at baseline to 0.534 ± 0.039 s at MTR80 (F(4, 88) = 17.94, *p* < 0.001, partial eta-squared = 0.449). Right stance time decreased from 0.744 ± 0.054 s at baseline to 0.662 ± 0.054 s at MTR80 (F(4, 84) = 24.39, *p* < 0.001, partial eta-squared = 0.537). Right swing time showed a smaller condition effect (F(4, 84) = 7.32, *p* < 0.001, partial eta-squared = 0.258). These temporal changes are consistent with the shorter gait-cycle duration accompanying faster walking.

### 3.6. Step Length and Step Time Asymmetry

Step length and step time asymmetry did not show meaningful condition effects. Signed step-length symmetry index did not show a significant five-condition effect (F(4, 88) = 1.37, *p* = 0.251) or activation-goal dose-response trend (0.178 percentage points per 20-percentage-point increase in activation goal, *p* = 0.106). Because step length was derived as treadmill speed multiplied by step time, signed step-time symmetry index was mathematically equivalent and was not tested separately. Thus, the hypothesis that unilateral right-limb feedback would systematically alter step length or step time asymmetry was not supported.

### 3.7. Ground-Reaction-Force Profiles

Force-profile analysis showed that the response was not limited to late-stance propulsion. Baseline summaries used 22 participants, whereas active-condition dose-response coefficients used 23. Anterior–posterior braking peak changed from −0.153 ± 0.028 N/BW at baseline to −0.246 ± 0.033 N/BW at MTR80, and propulsion peak increased from 0.164 ± 0.029 to 0.240 ± 0.041 N/BW. The vertical impact/loading peak increased from 1.029 ± 0.127 to 1.324 ± 0.137 N/BW. Early vertical loading slope increased from 0.127 ± 0.031 to 0.233 ± 0.074 N/BW/% gait cycle, and the first vertical peak occurred earlier at higher activation goals. Early-stance outward mediolateral peak force also increased, although this response was smaller than the anterior–posterior and vertical changes.

Time-based force measures supported this interpretation. Anterior–posterior braking impulse increased from 0.0288 ± 0.0055 N/BW·s at baseline to 0.0400 ± 0.0065 N/BW·s at MTR80, and propulsive impulse increased from 0.0311 ± 0.0040 to 0.0391 ± 0.0086 N/BW·s. Dose-response coefficients were 0.00133 N/BW·s per 20-point goal increase for braking impulse (false-discovery-rate-adjusted *p* = 0.020) and 0.00127 N/BW·s for propulsive impulse (adjusted *p* = 0.015). Net anterior–posterior impulse did not show a significant dose-response trend. Average vertical loading rate increased from 5.59 ± 1.34 to 10.44 ± 2.59 N/BW/s, and peak vertical loading rate increased from 12.81 ± 3.50 to 26.23 ± 9.01 N/BW/s. Vertical stance impulse decreased from 0.592 ± 0.040 to 0.550 ± 0.046 N/BW·s as stance time shortened.

**Figure 2:**
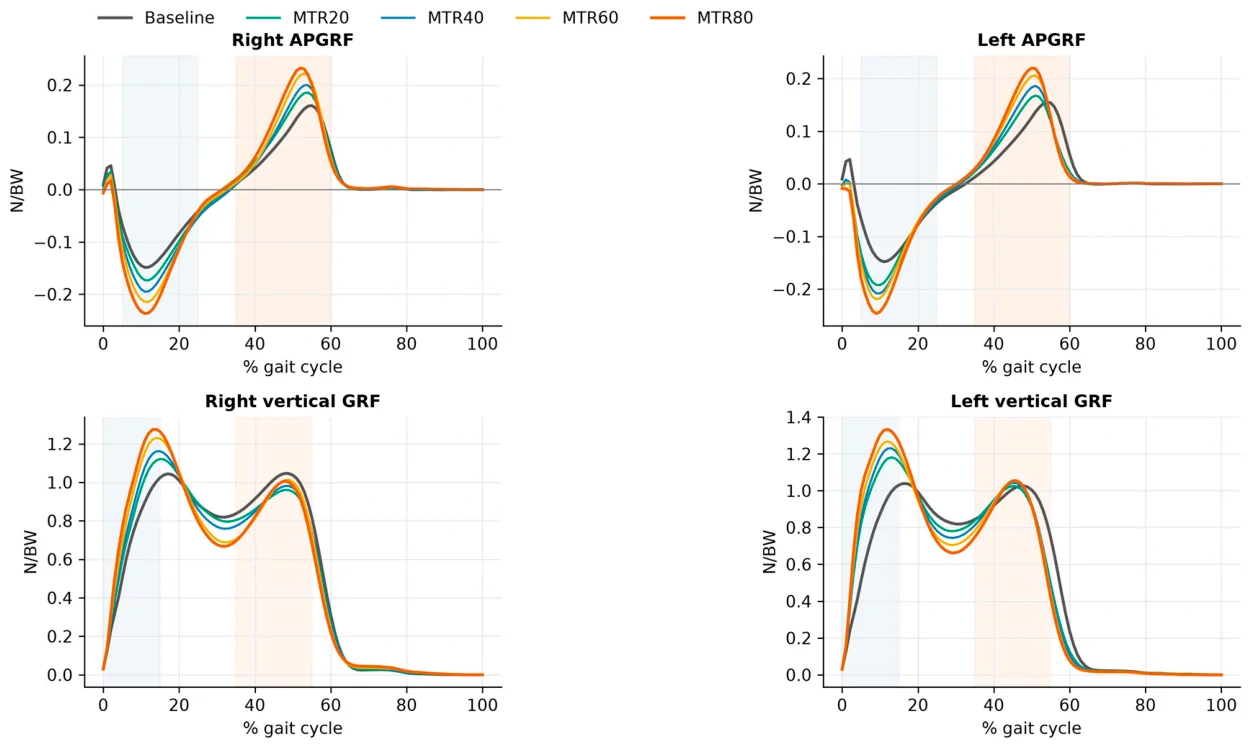
Full anterior–posterior and vertical ground-reaction-force profiles across the gait cycle. Shaded regions indicate the braking/loading and propulsion windows used for force-profile measures. Baseline profiles include 22 participants. Active-condition profiles include 23 participants.

**Figure 3:**
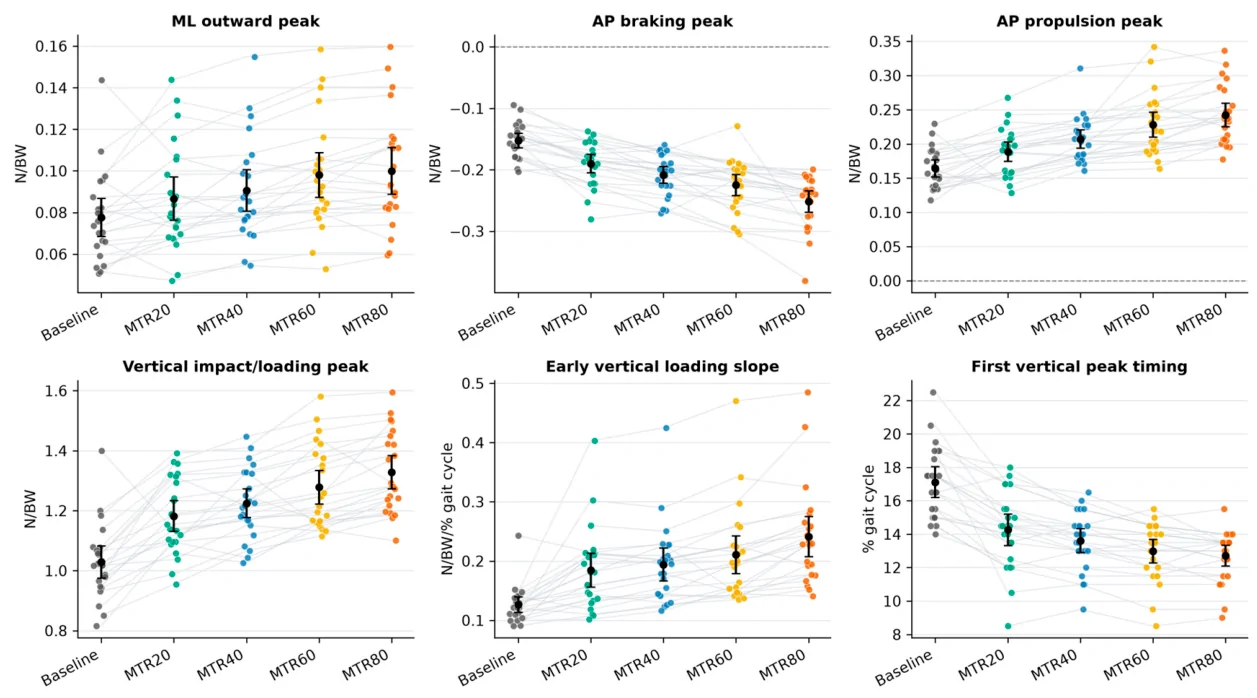
Stance-phase measures in three ground-reaction-force directions. Panels show early-stance side-normalized outward mediolateral peak, anterior–posterior braking peak, anterior–posterior propulsion peak, vertical impact/loading peak, early vertical loading slope (N/BW/% gait cycle), and first vertical peak timing. Dots show individual participants, gray lines connect repeated observations, and black markers show condition means with 95% confidence intervals. Baseline values include 22 participants; active-condition values include 23 participants.

The three ground-reaction-force directions showed different response patterns. The anterior–posterior direction carried the primary mechanical response, with larger early braking, larger late propulsion, and larger braking and propulsive impulses. The vertical direction changed mainly during early stance, with greater loading, steeper time-based loading rates, and earlier timing of the first vertical peak, while vertical stance impulse decreased as stance time shortened. The mediolateral direction showed a smaller and more selective response. Side-normalized outward mediolateral peak force during early stance increased by 0.0047 N/BW per 20-point goal increase (bootstrap 95% CI: 0.0033 to 0.0064; *p* < 0.001). Among the 22 participants with baseline waveforms, the baseline-to-MTR80 increase was 0.0201 N/BW. In contrast, mediolateral outward impulse did not show a significant dose-response trend after false-discovery-rate correction. Thus, the third force direction suggests a modest early-stance lateral-loading component during higher activation-goal walking, but not a consistent increase in integrated mediolateral loading. The full anterior-posterior and vertical profiles are shown in Figure 2, and the selected phase-specific measures in all three directions are shown in Figure 3.

Speed-adjusted participant fixed-effect models were examined in the 23-participant force sample. Activation goal and achieved speed were strongly collinear within participants (r = 0.975; variance-inflation factor = 20.6), so the models were interpreted as exploratory decomposition. Loading-response anterior–posterior GRF retained an activation-goal association after speed (beta = −0.0157, clustered *p* < 0.001, false-discovery-rate-adjusted *p* = 0.003), as did loading-response vertical GRF (beta = 0.0603, clustered *p* = 0.005, adjusted *p* = 0.030). Pre-swing vertical GRF (beta = −0.0390, clustered *p* = 0.027, adjusted *p* = 0.080), terminal-stance anterior–posterior GRF (beta = 0.0131, clustered *p* = 0.047, adjusted *p* = 0.114), and pre-swing mediolateral outward force (beta = −0.0035, clustered *p* = 0.019, adjusted *p* = 0.076) were nominally significant but did not survive correction. Peak propulsion was attenuated after achieved speed was added to the model (β = 0.0136 N/BW per 20-percentage-point increase, participant-clustered 95% CI: −0.0066 to 0.0337, *p* = 0.186, partial R^2^ = 0.048). Complete phase-by-direction results are provided in Table 2.

Exploratory variability analyses did not show broad evidence that higher activation goals increased step-to-step variability. Variability in propulsion, braking, step time, and step-time asymmetry did not show significant activation-goal dose-response trends. The primary interpretation therefore remains a graded shift in gait mechanics rather than a generalized increase in gait variability.

## 4. Discussion

Higher activation-goal conditions were accompanied by large, coordinated changes in walking speed, bilateral propulsion, braking magnitude, and step length, together with phase-specific changes in anterior–posterior and vertical ground-reaction-force profiles. Importantly, these changes occurred without systematic group-level step-length or step-time asymmetry. The findings for walking speed, propulsion, braking magnitude, step length, and asymmetry were consistent across the planned repeated-measures models, Greenhouse–Geisser checks, nonparametric tests, bootstrap intervals, and leave-one-participant-out analyses.

The walking speed response was large and graded. Participants increased speed by approximately 0.08 m/s at MTR20, 0.17 m/s at MTR40, 0.27 m/s at MTR60, and 0.37 m/s at MTR80. This progression was an intended functional component of the task: Participants were instructed to increase right plantar-flexor push-off and belt speed was selected using participant comfort and the app-displayed success rate. The result therefore describes an integrated activation-goal walking state rather than a speed-independent effect of medial gastrocnemius activation. The graded pattern is important because future clinical use of EMG-BFB will require reasonable activation-goal selection. In patient populations, appropriate feedback difficulty will likely depend on baseline walking speed, plantar-flexor capacity, and the ability to maintain the task without undesirable compensations.

The condition-by-order interaction also provided insight into how participants implemented the activation goals. Participants assigned to CDAB completed MTR60 and MTR80 first, followed by MTR20 and MTR40. This sequence produced the largest increase in walking speed and greatest reduction in step time. Participants assigned to BCDA began with MTR40, progressed through MTR60 and MTR80, and finished with MTR20; their speed and step-time responses were intermediate. Participants assigned to ABCD progressed conventionally from MTR20 through MTR80 and demonstrated the largest numerical increases in propulsion, braking magnitude, and step length. However, these numerical differences were descriptive and were not interpreted as sequence effects. Finally, participants assigned to DABC encountered MTR80 first and then completed MTR20, MTR40, and MTR60. This sequence produced the smallest graded speed and step-time responses. The condition-by-order interactions were significant for walking speed (*p* < 0.001) and step time (*p* = 0.015), but not for propulsion (*p* = 0.930), braking magnitude (*p* = 0.431), or step length (*p* = 0.291). Post hoc comparisons showed that both responses were significantly greater for CDAB than DABC, although CDAB did not differ significantly from ABCD or BCDA. The sequence-related differences were therefore expressed through walking speed and step timing rather than through sequence-specific changes in the measured external ground-reaction-force or step-length responses.

Although the study was not designed to determine an optimal sequence, these findings identify activation-goal progression as a potentially important biofeedback parameter. The pattern is consistent with evidence that the nervous system can regulate temporal and spatial components of gait partly independently and that practice schedule can influence the mechanical strategy used during locomotor adaptation [42,43]. It is also broadly compatible with the Challenge Point Framework, which proposes that the functional difficulty of practice depends on the interaction between task demands and performer skill [44]. Because retention and transfer were not assessed, these findings should be viewed as informing future comparisons of target-progression strategies rather than identifying an optimal sequence.

The propulsion results show that unilateral medial gastrocnemius feedback was accompanied by a bilateral whole-gait response. Although feedback was delivered from the right limb, both right and left peak propulsion increased. Participants therefore did not simply increase a local EMG signal in isolation; they adopted a faster walking pattern with longer steps and larger anterior–posterior force excursions. This pattern shows that the local feedback target was accompanied by changes in global gait mechanics.

The increase in braking magnitude, impulse, and force-profile measures helps explain the mechanical pattern. Faster walking and longer steps require greater control of forward body progression during early stance and greater propulsion during late stance. Higher activation-goal walking involved larger early AP braking, larger late stance AP propulsion, higher early vertical loading, earlier timing of the first vertical GRF peak, and a lower midstance vertical minimum. Thus, the response was not just a taller propulsion peak, it was a coordinated stance-phase adaptation involving load acceptance, braking, vertical support, and push-off. The normalized-waveform loading slope and vertical peak timing are important because they indicate how quickly and when the limb accepted body weight, while the AP braking and propulsion panels show how the body first decelerated and then reaccelerated the center of mass during stance.

The impulse and time-based loading-rate results clarify the distinction between amplitude, timing, and integrated force. Larger braking and propulsive impulses indicate that the AP peak changes were accompanied by greater time-integrated braking and push-off forces, consistent with prior work linking AP impulse to walking speed and propulsion mechanics [7,9]. In contrast, net AP impulse changed little because braking and propulsion cancel when integrated across stance. The vertical results showed a different pattern. Loading peaks and loading rates increased while vertical stance impulse decreased as stance time shortened. This is biomechanically plausible for faster treadmill walking and indicates faster load acceptance rather than greater total vertical support impulse [35,37]. These force-time measures should be interpreted as external mechanical demands. They are consistent with greater early-stance force-acceptance demand and greater late-stance push-off demand.

The mediolateral findings add a useful but more cautious third-direction interpretation. The outward mediolateral peak increased modestly during early stance, whereas mediolateral impulse did not. This pattern suggests that participants may have accepted weight with slightly larger lateral force excursions at higher activation goals, but the absence of an impulse effect argues against a large global change in mediolateral loading across stance. These findings should not be interpreted as direct evidence of improved or impaired balance, because the study did not include perturbations, margin-of-stability outcomes, center-of-mass kinematics, or overground obstacle negotiation. Instead, they indicate that increasing medial gastrocnemius feedback goals altered how participants organized stance-phase forces in three directions, with anterior–posterior propulsion and braking as the dominant effect, vertical loading as a coupled support response, and mediolateral loading as a smaller secondary adaptation.

The speed-adjusted analyses also clarify the interpretation. Loading-response anterior–posterior and vertical forces retained activation-goal associations after achieved speed and false-discovery-rate correction, whereas peak propulsion and the remaining phase variables did not. Because activation goal and achieved speed were highly collinear, these models refine but do not replace the primary intervention analysis and cannot identify an isolated causal feedback effect. They instead indicate that the graded activation-goal walking response included early-stance force changes that were not fully represented by achieved speed alone. The exploratory variability findings further suggest that these changes were not accompanied by a broad increase in step-to-step inconsistency.

The EMG findings support the interpretation that participants responded to the intended activation-goal task. Delsys medial gastrocnemius amplitude increased across activation-goal levels, although between-participant variability was substantial. Trial-matched mTrigger and Delsys amplitudes were moderately positively associated after within-participant centering, indicating that the systems captured related directional changes but should not be treated as interchangeable measures.

The asymmetry finding was contrary to our hypothesis. We expected that right-limb feedback might alter step length or step time asymmetry. Instead, participants increased speed, propulsion, braking magnitude, and step length without a consistent group-level asymmetry effect. This suggests that healthy adults were generally able to meet the unilateral feedback task by changing the overall walking state rather than by developing a systematic right-left stepping bias. In clinical populations it would still be important to monitor both feedback success and gait quality so that increases in activation or speed are not achieved through undesirable asymmetry.

These findings are consistent with prior studies showing that biofeedback can modify propulsion-related gait mechanics [20,21,22]. The present study extends that work by characterizing graded biomechanical responses during portable auditory EMG-BFB with individualized activation goals. Understanding how portable EMG biofeedback reshapes gait mechanics may inform future strategies to address pathological gait, improve walking after neurologic injury, support mobility in older adults, and enhance propulsion-related performance in healthy individuals. Future work should examine overground walking, longer-duration training, retention, and individualized goal progression.

### Limitations

This study has several limitations. The sample included healthy adults, and each activation-goal condition involved a short treadmill trial; rehabilitation efficacy, retention, and overground transfer were not tested. Belt speed was individualized to support activation-target attainment, so the study does not separate activation-goal effects from the accompanying speed progression. Counterbalancing balanced sequence assignment, but it did not eliminate the observed sequence dependence. Learning, fatigue, or carryover therefore cannot be excluded. No sham-feedback or no-feedback control condition was included. Therefore, the specific contribution of auditory feedback could not be separated from task instruction, attention, motivation, or repeated exposure. Responses may also differ during community walking, where visual scanning, obstacle negotiation, and turning impose additional demands.

## 5. Conclusions

During portable auditory EMG biofeedback of the right medial gastrocnemius, higher activation-goal conditions were completed at progressively faster treadmill speeds and were accompanied by changes in bilateral propulsion, braking magnitude, step length, and stance-phase ground-reaction-force profiles. Unilateral feedback was not associated with systematic step-length or step-time asymmetry. These results characterize the coordinated whole-gait response across increasing muscle-activation goals and provide a basis for future evaluation of portable EMG biofeedback in gait rehabilitation and performance settings.

## Figures and Tables

**Table 1:** Activation-goal dose-response coefficients across MTR20–MTR80.

Outcome	Coefficient (Bootstrap 95% CI)	t(22)	*p*	Cohen’s dz	FDR *p*
Walking speed	0.100 (0.093, 0.108) m/s	23.77	<0.001	4.96	<0.001
Mean bilateral peak propulsion	0.020 (0.016, 0.025) N/BW	8.44	<0.001	1.76	<0.001
Mean bilateral braking magnitude	0.022 (0.017, 0.026) N/BW	9.38	<0.001	1.96	<0.001
Mean step length	0.034 (0.030, 0.038) m	15.44	<0.001	3.22	<0.001
Mean step time	−0.017 (−0.021, −0.013) s	−8.17	<0.001	−1.70	<0.001
Early-stance outward ML peak	0.0047 (0.0033, 0.0064) N/BW	5.87	<0.001	1.22	<0.001
AP braking peak	−0.020 (−0.025, −0.015) N/BW	−8.44	<0.001	−1.76	<0.001
AP propulsion peak	0.018 (0.014, 0.023) N/BW	7.90	<0.001	1.65	<0.001
Vertical impact/loading peak	0.049 (0.034, 0.064) N/BW	6.39	<0.001	1.33	<0.001
Early vertical loading slope	0.0185 (0.0131, 0.0241) N/BW/% gait cycle	6.37	<0.001	1.33	<0.001
First vertical peak timing	−0.526 (−0.702, −0.370) % gait cycle	−6.06	<0.001	−1.26	<0.001
AP braking impulse	0.0013 (0.0004, 0.0024) N/BW·s	2.61	0.016	0.54	0.020
AP propulsive impulse	0.0013 (0.0004, 0.0022) N/BW·s	2.81	0.010	0.59	0.015
Vertical stance impulse	−0.016 (−0.021, −0.011) N/BW·s	−5.91	<0.001	−1.23	<0.001
Average vertical loading rate	0.968 (0.756, 1.185) N/BW/s	8.54	<0.001	1.78	<0.001
Peak vertical loading rate	2.664 (1.974, 3.398) N/BW/s	7.20	<0.001	1.50	<0.001

Note: Coefficients are per 20-percentage-point increase in activation goal and were calculated from the four active conditions in 23 participants. The 22-participant baseline summaries and 20-participant cross-system comparison reported in the text are not included in Table 1. Primary gait-outcome *p* values are from planned unadjusted slope tests, and the FDR column repeats those values for the primary rows. Benjamini–Hochberg correction was applied within the declared exploratory GRF peak/window and force-time families. AP, anterior–posterior; ML, mediolateral; GRF, ground reaction force.

**Table 2:** Speed-adjusted phase-by-direction ground-reaction-force models.

Gait Phase	GRF Direction	Goal Coefficient (Bootstrap 95% CI)	Partial R^2^	*p*	FDR *p*
Loading response	APGRF	−0.016 (−0.025, −0.0094)	0.125	<0.001	0.003
Loading response	Vertical GRF	0.060 (0.022, 0.103)	0.099	0.005	0.030
Loading response	ML outward	0.0029 (−0.0017, 0.0076)	0.019	0.261	0.448
Midstance	APGRF	−0.0036 (−0.011, 0.0051)	0.014	0.441	0.588
Midstance	Vertical GRF	−0.0045 (−0.038, 0.019)	0.002	0.778	0.849
Midstance	ML outward	−0.0036 (−0.0083, 0.0006)	0.037	0.150	0.299
Terminal stance	APGRF	0.013 (0.0028, 0.027)	0.080	0.047	0.114
Terminal stance	Vertical GRF	−0.0078 (−0.038, 0.032)	0.003	0.684	0.821
Terminal stance	ML outward	−0.0032 (−0.0081, 0.0030)	0.030	0.304	0.456
Pre-swing	APGRF	−0.0003 (−0.015, 0.012)	0.000	0.967	0.967
Pre-swing	Vertical GRF	−0.039 (−0.072, −0.010)	0.068	0.027	0.080
Pre-swing	ML outward	−0.0035 (−0.0061, −0.0007)	0.079	0.019	0.076

Note: Each model included 114 participant-condition observations from 23 participants and specified participant fixed effects, activation goal per 20-percentage-point increase, and achieved speed as a continuous predictor. Coefficients are in N/BW. Confidence intervals use 5000 participant-level bootstrap resamples; *p* values use participant-clustered covariance; FDR *p* values use Benjamini–Hochberg correction across the 12 activation-goal terms. Partial R^2^ is the activation-goal effect size calculated from nested-model residual sums of squares. AP, anterior–posterior; ML, side-normalized outward mediolateral; GRF, ground reaction force.

## Data Availability

Deidentified analysis-ready data and associated processing code are available on GitHub https://github.com/rkoiler/portable-emg-biofeedback-gait-data (accessed on 17 August 2026) and archived in Zenodo https://doi.org/10.5281/zenodo.21797497.
